# Poor Unstable Midgut Microbiome of Hard Ticks Contrasts With Abundant and Stable Monospecific Microbiome in Ovaries

**DOI:** 10.3389/fcimb.2020.00211

**Published:** 2020-05-08

**Authors:** Melina Garcia Guizzo, Saraswoti Neupane, Matej Kucera, Jan Perner, Helena Frantová, Itabajara da Silva Vaz, Pedro L. de Oliveira, Petr Kopacek, Ludek Zurek

**Affiliations:** ^1^Central European Institute of Technology (CEITEC), Center for Zoonoses, University of Veterinary and Pharmaceutical Sciences, Brno, Czechia; ^2^Biology Centre, Institute of Parasitology, Czech Academy of Sciences, Ceske Budejovice, Czechia; ^3^Department of Entomology, Kansas State University, Manhattan, KS, United States; ^4^Faculty of Science, University of South Bohemia, Ceske Budejovice, Czechia; ^5^Laboratório de Imunologia Aplicada a Sanidade Animal, Centro de Biotecnologia, Universidade Federal do Rio Grande do Sul, Porto Alegre, Brazil; ^6^Faculdade de Veterinária, Universidade Federal do Rio Grande do Sul, Porto Alegre, Brazil; ^7^Laboratório de Bioquímica de Artrópodes Hematófagos, Instituto de Bioquímica Médica, Universidade Federal do Rio de Janeiro, Rio de Janeiro, Brazil; ^8^Department of Chemistry and Biochemistry, Mendel University, Brno, Czechia

**Keywords:** tick, *Ixodes ricinus*, *Rhipicephalus microplus*, midgut microbiome, ovary microbiome, symbiosis, *Midichloria mitochondrii*

## Abstract

Culture-independent metagenomic methodologies have enabled detection and identification of microorganisms in various biological systems and often revealed complex and unknown microbiomes. In many organisms, the microbiome outnumbers the host cells and greatly affects the host biology and fitness. Ticks are hematophagous ectoparasites with a wide host range. They vector a number of human and animal pathogens and also directly cause major economic losses in livestock. Although several reports on a tick midgut microbiota show a diverse bacterial community, in most cases the size of the bacterial population has not been determined. In this study, the microbiome was quantified in the midgut and ovaries of the ticks *Ixodes ricinus* and *Rhipicephalus microplus* before, during, and after blood feeding. Although the size of bacterial community in the midgut fluctuated with blood feeding, it was overall extremely low in comparison to that of other hematophagous arthropods. In addition, the tick ovarian microbiome of both tick species exceeded the midgut 16S rDNA copy numbers by several orders of magnitude. This indicates that the ratio of a tick midgut/ovary microbiome represents an exception to the general biology of other metazoans. In addition to the very low abundance, the tick midgut diversity in *I. ricinus* was variable and that is in contrast to that found in the tick ovary. The ovary of *I. ricinus* had a very low bacterial diversity and a very high and stable bacterial abundance with the dominant endosymbiont, *Midichloria* sp. The elucidation of this aspect of tick biology highlights a unique tissue-specific microbial-invertebrate host interaction.

## Introduction

Ticks are obligate blood-feeding ectoparasites of a wide range of hosts including wild and domestic animals and people. They transmit a large number of pathogens and also directly cause great economic losses in livestock (Estrada-Peña, 2015). While the majority of studies focused on detection of the pathogens ticks transmit, several studies also explored the overall microbial diversity of ticks, including that of *Ixodes ricinus* and *Rhipicephalus microplus* (Andreotti et al., 2011; Carpi et al., 2011; Greay et al., 2018). However, the identification of the microbial taxa is only the first step toward the understanding of host-microbiota interactions. Till present, the main advance has been made in the functional characterization of endosymbionts housed in the tick ovary. Maternally-inherited bacteria *Coxiella* sp. and *Francisella* sp. were shown to be essential for tick molt and/or survival (Guizzo et al., 2017; Duron et al., 2018). In contrast, little is known about the microbiota residing in the tick midgut, its role in tick physiology and vector competence for pathogens as well as its potential origins (Narasimhan et al., 2014; Narasimhan and Fikrig, 2015; Vayssier-Taussat et al., 2015; Bonnet et al., 2017). In the past decade, several metagenomic studies using next-generation sequencing (NGS) have described the tick midgut as an organ harboring a diverse bacterial community (Moreno et al., 2006; Andreotti et al., 2011; Budachetri et al., 2014; Clayton et al., 2015). However, it is important to point that NGS methods are based on an amplification of the bacterial 16S rDNA gene fragment, allowing the detection of less abundant bacteria, but also potentially introduce false-positive results due to background contamination and also do not distinguish between viable bacteria and pieces of DNA from lysed cells (Goodrich et al., 2014; Huttenhower et al., 2014; Strong et al., 2014). Moreover, it was demonstrated that the sterilization protocols of the tick surface greatly impact results of the microbial diversity (Binetruy et al., 2019).

This study aimed to assess the bacterial diversity in the midgut and ovaries of *I. ricinus*, and to investigate its potential source from the host blood and skin by culture-independent approaches. Moreover, the dynamic of bacterial colonization was elucidated through the absolute quantification of the microbiome in the midgut and ovaries of *I. ricinus* and *R. microplus* during the feeding course on the vertebrate host.

## Materials and Methods

### Tick Rearing

Wild-caught unfed *I. ricinus* females were collected by flagging on grass around Ceske Budejovice, Czech Republic. Ticks were maintained in the rearing facility at the Institute of Parasitology, Biology Centre Czech Academy of Sciences (CAS) under controlled conditions (temperature: 24°C and humidity: 95%). To complete the life cycle, unfed female ticks were allowed to feed on guinea pigs until the natural detachment from the host. All laboratory animals were treated in accordance with the Animal Protection Law of the Czech Republic No. 246/1992 Sb., ethics approval No. 25/2018. The study was approved by the Institute of Parasitology, Biology Centre CAS and Central Committee for Animal Welfare, Czech Republic (Protocol No. 1/2015).

*Rhipicephalus microplus* (Porto Alegre strain) were maintained on Hereford cattle brought from a naturally tick-free area (Santa Vitória do Palmar, Brazil; 33° 32′2″ S, 53° 20′59″ W) and kept in individual tick-proof pens on a slatted floor at the Faculdade de Veterinária of Universidade Federal do Rio Grande do Sul (Brazil) (Reck et al., 2009). The colony is maintained without the introduction of field ticks. Calves were infested with 15-day-old tick larvae. Partially engorged and fully engorged females were collected directly from the host and after natural detachment, respectively. All animal care and experimental protocols were conducted following the guidelines of the institutional care and use committee (Ethics Committee on Animal Experimentation) and were approved under the registry 14403/protocol 07.

### Vertebrate Host and Tick Samples

*Ixodes ricinus* females were collected at several time points during a blood-feeding course on a guinea pig host. Unfed ticks, ticks on day 1, 3, and 5 during blood feeding, fully fed ticks, and ticks on day 2 and 6 after natural detachment were dissected to remove the midgut. Ovaries were dissected out from fully fed ticks and from ticks on day 2 and 6 after detachment. Similarly, the midgut and ovaries from partially engorged (mean weight 75.0 mg/tick) and fully engorged females were dissected from *R. microplus*. Before dissection, ticks were surface sterilized with 70% ethanol for 1 min, followed by three washes in sterile phosphate buffered saline (PBS) (Sigma, St. Louis, Missouri). *Ixodes ricinus* and *R. microplus* were then subjected to the 16S rDNA gene absolute quantification through qPCR and *I. ricinus* to Illumina sequencing analysis.

Swab skin and blood samples were collected from the vertebrate host (guinea pig). The bacterial recovery from the host skin was performed through the homogenization of the swab skin in 500 μl of sterile PBS. Before blood collection, the host skin was cleaned with 70% ethanol and blood was collected from the jugular vein using a sterile syringe and needle.

### Genomic DNA Extraction and Culture-Independent Analysis of the Bacterial Community in the Midgut and Ovaries of *I. ricinus*

The tick genomic DNA was isolated using the PowerSoil DNA isolation kit (MO BIO, Hilden, Germany) according to the manufacturer's instructions. The bacterial community of the midgut and ovary of wild *I. ricinus*, the midgut of laboratory-reared *I. ricinus*, host swab skin, and host blood was investigated by the 16S rDNA sequencing of the total DNA. For the midgut DNA extraction, the whole organ was used, including the lumen content. The protocol was adapted for fully fed females using a double volume of reaction adjusted at the final step. The sequencing library preparation was done at the Integrated Microbiome Resource (IMR) facility, Dalhousie University, Halifax, Nova Scotia, Canada as described by Comeau et al. (2017). Briefly, a dual-indexing one-step PCR strategy was applied using the extracted total DNA as a template to amplify the V6-V8 16S rDNA region (Comeau et al., 2011) with the Illumina Nextera adaptors plus the indices/barcodes. DNA-free water (Top-Bio, Praha, Czech Republic) was used as a negative control. PCR was performed in duplicates using two template dilutions, combined in one plate and visualized on an Invitrogen 96-well E-gel (Invitrogen, California, USA). Amplicons were cleaned-up and normalized using the high-throughput Invitrogen Sequal-Prep 96-well plate kit (Invitrogen, California, USA). Using the Invitrogen Qubit double-stranded DNA high sensitivity fluorescence-based method (Invitrogen, California, USA), the samples were pooled and quantified before the sequencing on an Illumina MiSeq machine.

The raw paired-end sequence reads obtained from the Illumina MiSeq sequencing platform were analyzed using the Mothur bioinformatic software pipeline (version 1.42.3) (Schloss et al., 2009). In short, primers were removed, and paired-end sequences were assembled into contigs. The quality of each contig (sequence hereafter) was checked. Sequence with low quality (*q* < 25), ambiguous base and ambiguous length (<200 and >450 bp) sequences were removed. High quality sequences were aligned with SSU rRNA SILVA reference alignment (Yilmaz et al., 2014) using Needleman-Wunsch global alignment algorithm (Needleman and Wunsch, 1970). VSEARCH (Rognes et al., 2016) was used to check chimera and removed chimeric sequences if present. High quality, non-chimeric sequences were clustered into operational taxonomic units (OTUs) with 97% sequence similarity using distance-based greedy cluster (DGC) algorithm. The representative sequence for each OTU was used to determine the phylogenetic identification of OTUs. These sequences were compared with SILVA reference datasets (Quast et al., 2013) using basic alignment search tool (BLAST) (Altschul et al., 1997). Erroneous OTUs (<2 sequence reads) were removed from the OTU table. Moreover, OTUs from background bacterial contamination were filtered from OTU table by removing OTUs that were clustered together with OTUs of the DNA-free water sample. The final OTU table was used for further statistical analyses.

### Quantification of the Bacterial 16S rDNA and Tick Elongation Factor Genes

The tick genomic DNA was isolated as described above. The single copy gene for tick elongation factor-1α (*ef-1*α) was used as a reference for data normalization (Nijhof et al., 2009). Quantification of the bacterial 16S rDNA gene (Nadkarni et al., 2002) was carried out in a LightCycler® 480 (Roche, Basel, Switzerland) with 50 cycles of 95°C (10 s), 60°C (10 s), and 72°C (10 s) following an initial denaturation of 95°C (10 min). Each 20 μl reaction mixture contained 10 μl of FastStart universal probe master (Rox) (Roche), 10 pmol of each primer, 5 pmol of TaqMan probe (Nadkarni et al., 2002), 2 μl of genomic DNA, and 5 μl of DNA-free water (Top-Bio, Praha, Czech Republic). Quantification of *ef-1*α was performed on the same equipment as described above with 50 cycles of 95°C (10 s), 60°C (10 s) and 72°C (10 s) following an initial denaturation of 95°C (10 min). The amplification was performed with 12.5 μl of FastStart universal SYBR green master (Rox) (Roche), 10 pmol of each primer, 2 μl of genomic DNA, and DNA-free water (Top-Bio). In order to perform the absolute quantification, standard curves were plotted using serial dilutions (10^6^-10^2^) of a plasmid containing the fragment of a single copy tick specie-specific *ef-1*α gene or the bacterial 16S rDNA gene. The *ef-1*α gene for *I. ricinus* and the bacterial 16S rDNA gene were cloned into TOPO plasmid vector (Invitrogen, California, USA), while the *ef-1*α for *R. microplus* into the pGEM®-T easy (Promega, Wisconsin, USA). Bacterial 16S rDNA counts in DNA-free water (Top-Bio, Praha, Czech Republic) were used as a control for background contamination. Midgut and ovary from seven *I. ricinus* and six *R. microplus* ticks were analyzed at each time-point during a feeding course on a vertebrate host. The total 16S rDNA copy number was represented either by an organ or by the tick elongation factor gene. Primers and probe sequences are listed in Table 1.

**Table 1:** List of primers and probe used in this study.

**Primer or probe**	**Sequence**	**Size of the amplicon**	**Tm (^**°**^C)**	**References**
V6-V8_For (B969F)	A CGC GI-1N RAA CCT TAC C	400 bp	53	Comeau et al., 2011
V6-V8_Rev (BA1406R)	AC GGG CRG TGW GTR CAA		57	
16S_For	TCC TAC GGG AGG CAG CAG T	466 bp	59	Nadkarni et al., 2002
16S_Rev	GGA CTA CCA GGG TAT CTAATC CTG TT		58	
16S_probe	CGT ATT ACC GCG GCT GCT GGC AC		70	
RmEF_For	CGT CTA CAA GAT TGG TGG CAT T	108 bp	60	Nijhof et al., 2009
RmEF_Rev	CTC AGT GGT CAG GTT GGC AG		60	
IREF For	ACG AGG CTC TGA CGG AAG	81 bp	60	Nijhof et al., 2009
IREF Rev	CAC GAC GCA ACT CCT TCA C		60	

### Statistical Analysis

Statistical analysis of the microbiome quantification data was performed using the GraphPad Prism software (version 8). First, all the data were tested for normality using Shapiro-Wilk test. Differences among three or more groups were determined using Kruskal-Wallis for a non-parametric test. Other comparisons were performed using Mann Whitney test or unpaired *t*-test depending on the Gaussian distribution. The values were considered statistically significant with *p* < 0.05. The correlation coefficient was calculated using the Spearman's rank.

Statistical analysis of the microbiome data from 16S rDNA sequencing was performed in R v.3.4.4 (R Core Team, 2018). Alpha diversity (species richness, Shannon diversity index, and Pielou's evenness) were calculated using the vegan package in R (Oksanen et al., 2019). Prevalence of bacterial phyla and genera in individual samples were summarized in a bar plot.

## Results

### A High Midgut vs. Low Ovary Diversity in *I. ricinus*

**Figure 1:**
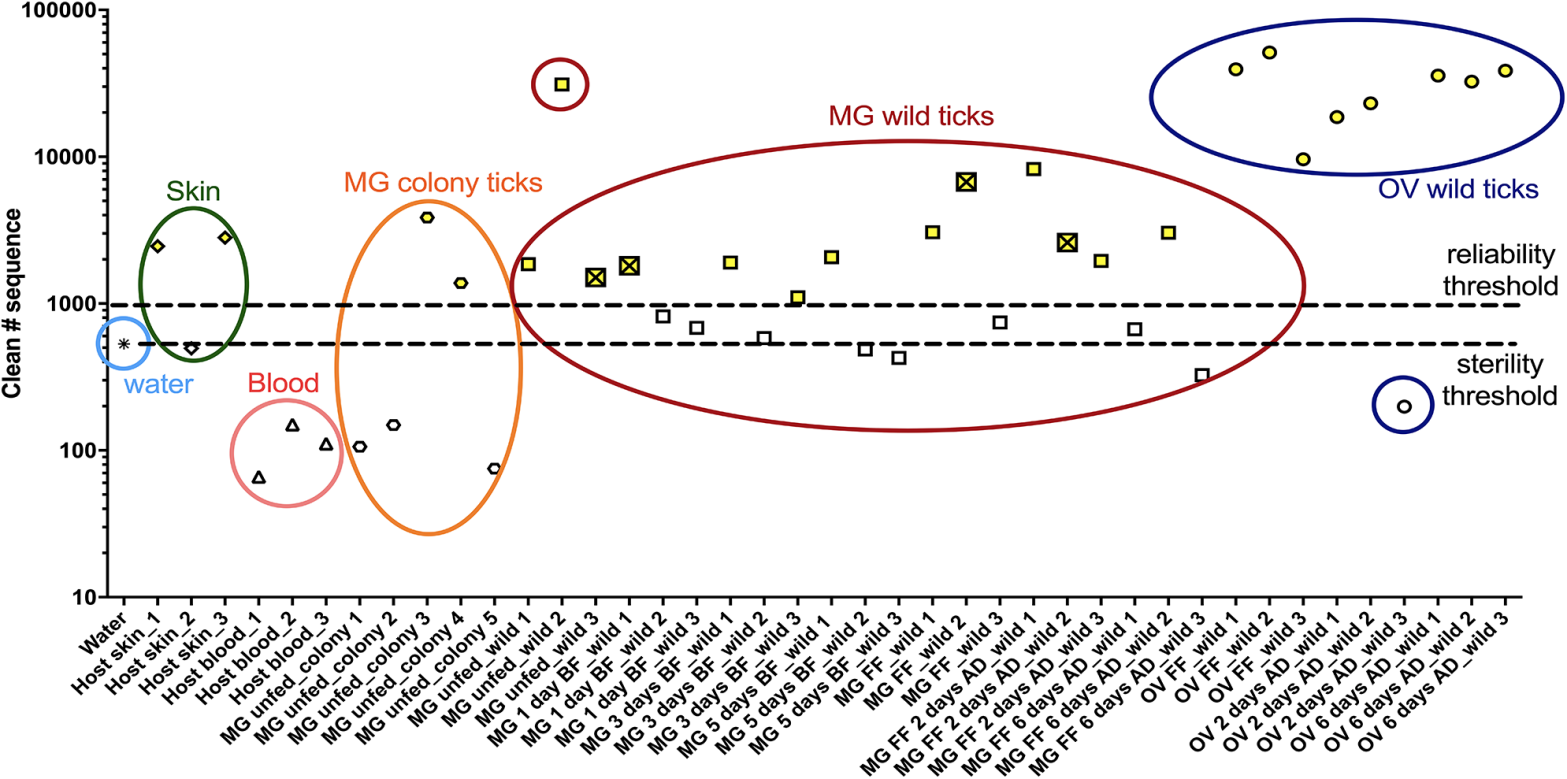
Numbers of good quality 16S rDNA gene sequences obtained through bioinformatic analysis. Samples above both thresholds (yellow) were selected to bacterial diversity analysis. The crossed square represents a tick infected with *Borreliella* sp. MG, midgut; OV, ovary; UF, unfed; 1D, 1-day; 3D, 3-days; 5D, 5-days; FF, fully fed; AD, after detachment.

In order to characterize the bacterial diversity of the *I. ricinus* midgut and ovary, the microbial community from wild and laboratory-reared ticks was investigated using Illumina sequencing. To elucidate the potential source, the microbiota from the host skin and blood was also assessed. The number of 16S rDNA good quality sequences obtained through bioinformatic analysis was used to determine two thresholds: sterility and reliability. The sterility threshold was used as a control for background contamination based on the bacterial counts obtained from DNA-free water (Top-Bio, Praha, Czech Republic). The reliability threshold was arbitrarily determined based on the number of good quality 16S rDNA sequences >1,000, which is directly related to the target DNA biomass and consequently to the DNA amplification. Only those samples above both thresholds were further analyzed (Figure 1 and Table S1).

**Figure 2:**
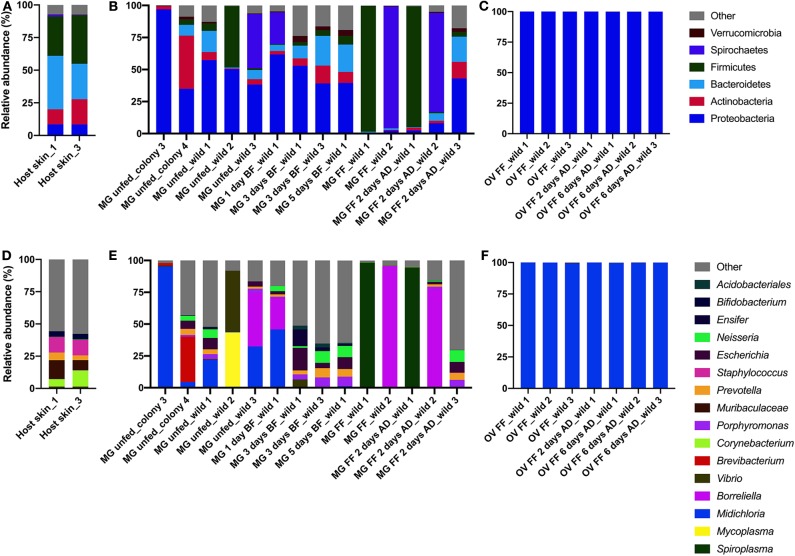
Bacterial diversity in the host skin **(A,D)** and the midgut **(B,E)** and ovary **(C,F)** of *Ixodes ricinus* on phylum **(A–C)** and genus **(D–F)** levels. MG, midgut; OV, ovary; BF, blood fed; FF, fully fed female; AD, after detachment.

A total of 300,206 good quality sequences were obtained and these were clustered into 1,138 OTUs at a similarity level 97% (Table 2 and Table S2). The bacterial diversity in the midgut was higher and variable comparing to that of the ovary, depending on the individual variation and the tick-borne pathogen infection status. Bacteria from the phyla Bacterioidetes and Proteobacteria, *Prevotella* sp., and *Neisseria* sp. respectively, were identified from the host skin (Figures 2A,D) as well as from midguts of all blood fed ticks (Figures 2B,E). However, any bacterial genus was found in all the midgut samples irrespective of the tick feeding status (Figure 2E and Table S2). As expected, only wild ticks were infected with tick-borne pathogens. Four out of twelve wild ticks were positive for *Borreliella* sp., the bacterial agent causing Lyme disease. *Spiroplasma* sp. was the dominant bacterial taxon in the midgut of two blood fed females. Other bacterial genera including *Escherichia* sp., *Neisseria* sp. and *Porphyromonas* sp. were found in a high frequency in the tick midgut (Figure 2E and Table S2). On average, more bacterial genera were found in the midgut of unfed wild ticks in comparison to that of the laboratory-reared ticks (Figure 2E and Table S2). The dominant bacterial taxon in ovaries was the endosymbiont *Midichloria* sp. (phylum Proteobacteria) regardless of the tick feeding stage (Figures 2C,F). These results are also reflected in the alpha diversity indices, including Shannon and Pielou's evenness, which are lower for the ovarian samples (Table 2). *Midichloria* sp. was also identified in the midgut from two unfed wild and two colony ticks, as well as in one blood fed tick (Figure 2E).

**Table 2:** Number of good quality sequences and operational taxonomic units (OTUs), and alpha diversity indices of the bacterial community in the ovary and the midgut of *Ixodes ricinus*.

**Source**	**Sample ID**	**# Sequences**	**# OTUs**	**Shannon diversity index**	**Pielou's evenness**
Skin	Host skin_1	2455	223	4.73	0.87
Skin	Host skin_3	2814	223	4.56	0.84
Ovary	OV FF_wild 1	39414	2	0.00	0.00
Ovary	OV FF_wild 2	51300	2	0.00	0.00
Ovary	OV FF_wild 3	9608	8	0.05	0.02
Ovary	OV 2 days AD_wild 1	18633	3	0.00	0.00
Ovary	OV 6 days AD_wild 1	35750	20	0.03	0.01
Ovary	OV 6 days AD_wild 2	32513	17	0.03	0.01
Ovary	OV 6 days AD_wild 3	38563	12	0.01	0.00
Ovary	MG unfed_colony 3	3857	13	0.29	0.11
Ovary	MG unfed_colony 4	1382	55	3.00	0.75
Midgut	MG unfed_wild 1	1855	103	3.74	0.81
Midgut	MG unfed_wild 2	31070	159	1.28	0.25
Midgut	MG unfed_wild 3	1505	29	1.83	0.54
Midgut	MG 1 day BF_wild 1	1808	54	2.12	0.53
Midgut	MG 3 days BF_wild 1	1902	118	3.90	0.82
Midgut	MG 3 days BF_wild 3	1105	93	4.13	0.91
Midgut	MG 5 days BF_wild 1	2068	111	4.14	0.88
Midgut	MG FF_wild 1	3065	16	0.50	0.18
Midgut	MG FF_wild 2	6743	41	0.37	0.10
Midgut	MG FF 2 days AD_wild 1	8245	48	0.79	0.20
Midgut	MG FF 2 days AD_wild 2	2602	79	1.45	0.33
Midgut	MG FF 2 days AD_wild 3	1949	113	4.19	0.89

*MG, midgut; OV, ovary; BF, blood fed; FF, fully fed; AD, after detachment*.

### Hard Ticks Have Very Low Levels of the Midgut Microbiota During Blood Feeding Stage

**Figure 3:**
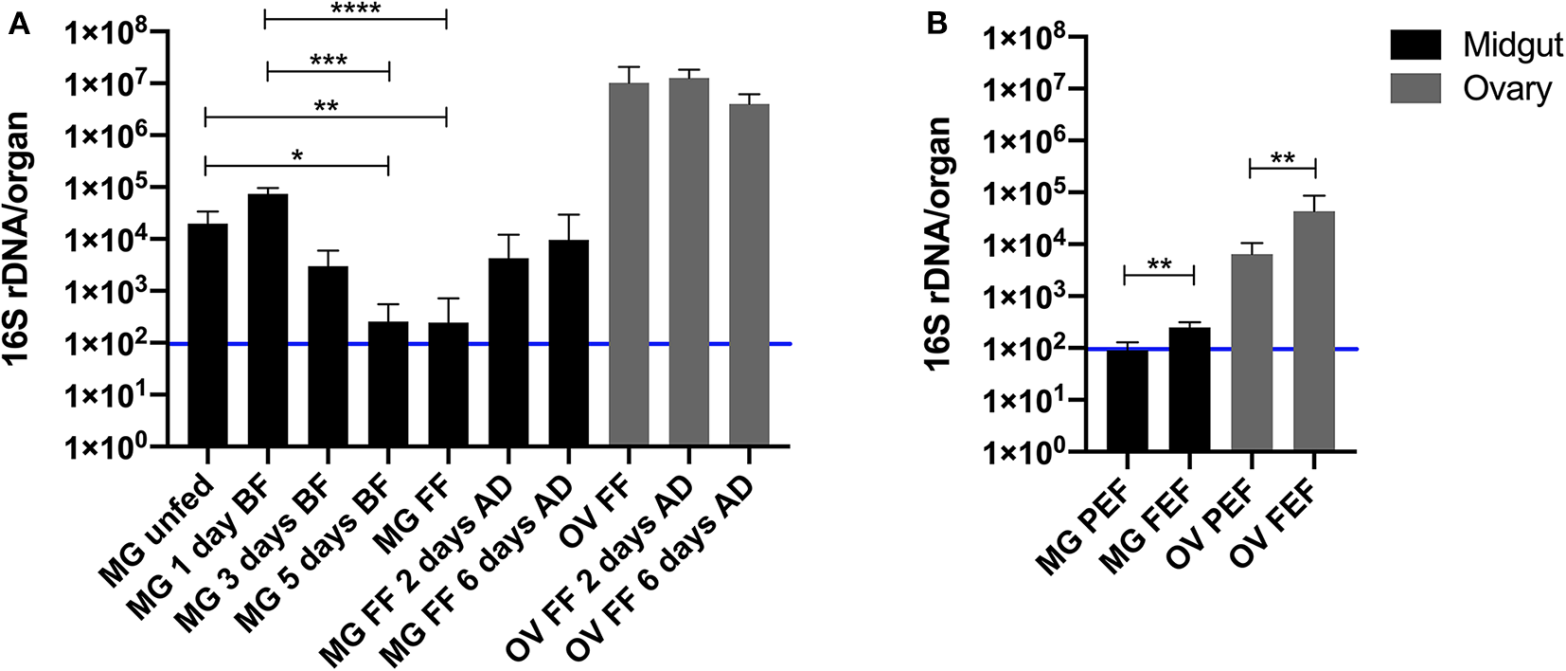
Quantification of the 16S rDNA gene per midgut (black) and ovary (gray) of *Ixodes ricinus* **(A)** and *Rhipicephalus microplus* **(B)** during blood feeding on a vertebrate host. MG, midgut; OV, ovary; BF, blood fed; FF, fully fed; AD, after detachment; PEF, partially engorged female; FEF, fully engorged female. The number of biological replicates analyzed in each time-point was 7 for *I. ricinus* and 6 for *R. microplus*. Error bars indicate standard deviation. The blue line represents the sterility threshold (16S rDNA counts in DNA-free water). Stars indicate statistically significant differences. *p < 0.05; **p < 0.01; ***p < 0.001; and ****p < 0.0001.

The size of the bacterial community in the midgut was followed through a feeding time course of *I. ricinus* wild females on guinea pigs. The total 16S rDNA level increased slightly but not significantly (*p* > 0.99) on day 1 of blood feeding and then started decline with statistically significant decreases on day 5 of blood-feeding (*p* = 0.03) and at the full engorgement (*p* = 0.009) (Figure 3A and Figure S1A). The total 16S rDNA level in the midgut negatively correlated (*r* = −0.88) with the tick weight during the feeding course (Figure S2).

Similarly to *I. ricinus*, very low levels of midgut microbiota were found in *R. microplus* revealing that only few bacterial cells were found in the whole digestive tract (~100 or less cells). However, due to nature of maintenance of this tick species on cattle, only two time points of blood feeding were examined in these ticks (Figure 3B and Figure S1C). The bacterial 16S rDNA gene copy number in the tick midgut during the blood feeding significantly increased (*p* = 0.002).

### Ovarian Microbial Abundance Greatly Exceeds That of the Midgut in Hard Ticks

In view of the low level of the total midgut microbiota in fully fed females of both, *I. ricinus* and *R. microplus*, we were interested in determination of assessment of the total microbial community in tick ovaries. The same fully fed female ticks from which the midguts were used for the total genomic DNA isolation had the ovarian DNA extracted and subjected to the bacterial quantification. For both tick species, *I. ricinus* and *R. microplus*, the 16S rDNA gene copy numbers per ovary or per house keeping gene levels exceeded that of the midgut by several orders of magnitude (Figures 3A,B and Figures S1B,D). *Ixodes ricinus* had 2–5 times order of magnitude higher levels of the bacterial 16S rDNA copy number in the ovary than in the midgut when expressed per organ (Figure 3A) and even higher when expressed per the house keeping gene (Figure S1B). The 16S rDNA absolute number in the ovary of *I. ricinus* decreased comparing 2–6 days after tick detachment from the host (Figure S1B).

Interestingly, the level of the bacterial 16S rDNA copy number in the ovary of *R. microplus* significantly increased when represented per organ (*p* = 0.004) or the house keeping gene (*p* = 0.02) during the blood feeding (Figure 3B and Figure S1D).

## Discussion

The digestive tract of metazoans is typically colonized by a numerous and diverse microbial community with an important role in the host physiology and overall fitness and survival (Engel and Moran, 2013; Heintz-Buschart and Wilmes, 2018). Several exceptions to this rule have been recently reported including some microbiome-free arthropod taxa (Hammer et al., 2019); however, blood-feeding arthropods generally possess a gut microbiota that affects their vector competence for pathogens (Weiss and Aksoy, 2011; Dennison et al., 2014; Narasimhan et al., 2014; Narasimhan and Fikrig, 2015). For hematophagous arthropods, blood is the main source of nutrients and is essential for embryo development and molting of juvenile stages. The influx of blood into the digestive system typically leads to an expansion of the midgut bacterial community in blood feeding arthropods as shown, for example, for mosquitoes and triatomine bugs (Eichler and Schaub, 2002; Oliveira et al., 2011). In *Aedes aegypti* the blood uptake increased the culturable midgut bacterial population by about three orders of magnitude (from 10^3^ to 10^6^ per insect) (Oliveira et al., 2011). In the triatomine bug, *Rhodnius prolixus*, the level of the gut bacterial symbiont *Rhodococcus rhodnii* increased 15-fold in the fifth instars after 10 days of blood feeding and resulted in the population of 10^8^ bacteria per insect (Eichler and Schaub, 2002).

During the feeding course on a vertebrate host, hard ticks increase their size 100–1,000 times reflecting a great influx of blood which represents a nutritionally rich substrate for ticks, potentially available also for bacteria present in the tick midgut. Nevertheless, unlike reported for other hematophagous arthropods, we showed here that in the hard ticks, *I. ricinus* and *R. microplus*, the blood intake is not associated with bacterial growth in the midgut. In fact, our results show that there is a negative correlation between the amount of blood consumed and the midgut bacterial level in *I. ricinus*; 16S rDNA levels per organ ranged from 10^4^ in unfed females to 10^2^ in fully fed females. In *R. microplus*, due the one-host life cycle for all tick stages, the unfed female could not be obtained for analysis. The bacterial levels in this tick species, regardless the blood influx, remained very low with approximately 10^2^ 16S rDNA copies per midgut in partially and fully engorged females. In both cases, these bacterial levels were close to the sterility technical threshold. Taken together, the numbers do not allow to define if the few bacterial cells found in *I. ricinus* and *R. microplus* midgut are just a transient population or constitute a resident community.

Several hypotheses were formulated for the primary cause of low bacterial levels in the midgut of ticks. First, it is hypothesized that hemoglobin fragments generated during blood digestion and the complement system from the host blood (Fogaça et al., 1999; Nakajima et al., 2009; Kopáček et al., 2010) negatively impact the midgut bacterial community and cause its rapid decline. The second hypothesis is that the epithelial immunity regulates the bacterial population in the tick midgut by antimicrobial peptides and reactive oxygen species (ROS) (Sonenshine and Macaluso, 2017). For example, it was demonstrated in the *I. ricinus* midgut transcriptome that the immune genes such as defensin, lysozyme, and microplusin were substantially upregulated at the end of tick feeding (Perner et al., 2016). Moreover, the capillary feeding of *Dermacentor variabilis* with *Escherichia coli* resulted in expression of defensin-like peptides in tick midgut (Sonenshine et al., 2002). In the majority of hematophagous animals, the digestion is an extracellular process and takes place in the gut lumen. Ticks, however, represent an exception as they digest blood intracellularly in the specialized digestive midgut cells that take up blood components by means of massive endocytosis (Sojka et al., 2013). The third hypothesis is that the midgut microbiota is reduced in part, by the tick digestive cells. Some midgut microbiota might be endocytosed by the tick digestive epithelial cells along with the vertebrate blood components (Lara, 2005) and consequently digested.

Andreotti et al. (2011) reported that the *R. microplus* midgut harbors a highly diverse bacterial community belonging to 11 bacterial genera. Similarly, we showed here that the *I. ricinus* midgut carries a variable bacterial population that differs depending on individual ticks and the tick-borne pathogen infection status. The host skin as a potential source of bacteria in the midgut of *I. ricinus* is indicated by detecting the same bacterial taxa (e.g., *Prevotella* sp. and *Neisseria* sp.) from both, host skin and in the midgut, of blood fed females, regardless the time-point of the blood feeding course. *Prevotella* sp. has been identified as part of the healthy skin microbiome and *Neisseria* sp. is common in the mucosal surface of vertebrate hosts of *I. ricinus* (Elliott et al., 2005; Dimitriu et al., 2019). In contrast to the ovary, the midgut did not show a core microbiome, as we did not detect any common bacterial genera in all midgut samples. It is likely that most of identified bacteria in the midgut originated from the environment; they were accidentally ingested and constitute just a transient population. The hypothesis that the source of the midgut bacteria is the environment is further supported by the finding that wild ticks have a higher bacterial diversity than colony ticks maintained under laboratory-controlled conditions.

Bacterium from the genus *Midichloria* sp. found in the midgut of unfed and in 1-day blood fed *I. ricinus* corroborated the study of Olivieri et al. (2019). *Vibrio* sp. detected in the midgut of five out from fourteen ticks is not usually reported in metagenomic analysis of ticks and it likely represents a bacterium of the environmental origin. *Mycoplasma* sp. found in the midgut of one unfed *I. ricinus* has been reported from ticks previously (Hornok et al., 2019) but its significance in tick biology as well as from the clinical perspective is unknown. The midgut of two blood fed ticks were dominated by *Spiroplasma* sp. Different species in the genus *Spiroplasma* were described in arthropods and plants in commensal, mutualist, and pathogenic associations (Regassa and Gasparich, 2006). In ticks, this taxon was detected in several species and described as a facultative symbiont (Henning et al., 2006; Duron et al., 2017). Four out of twelve wild ticks were infected with a bacterium from the genus *Borreliella*, which make part of a complex of species that are vectored by ticks and cause Lyme disease in humans (Lane et al., 1991). *Borreliella* sp. are acquired by ticks during blood feeding from an infected host at the early tick life-stages and persist in the midgut after tick molting (Benach et al., 1987). Presence of *Borreliella* sp. in *I. ricinus* in some parts of Central Europe can be very high (Strnad et al., 2017) and it is therefore not surprising that it was found in the midgut of 33% of the wild ticks analyzed. It appears that both, *Spiroplasma* sp. and *Borreliella* sp., are capable of outcompeting other bacteria in the midgut during tick blood feeding and blood digestion and become the dominant member of the tick midgut microbiome. However, our data are limited and studies with larger number of specimen are needed to confirm these results.

In contrast to the tick midgut, we found an abundant and stable bacterial community with very low diversity in the ovary of *I. ricinus* with the dominant endosymbiont, *Midichloria* sp. This result corroborates with the finding of high levels of *Midichloria* sp. in fully fed *I. ricinus* females (Sassera et al., 2008). In the *R. microplus* ovary, it was shown that the dominant endosymbiont is *Coxiella* sp. representing 98.2% of the 16S rDNA sequences in fully engorged females (Andreotti et al., 2011). The presence of *Midichloria* sp. and *Coxiella* sp. in tick ovaries and eggs points out to vertical transmission, typical for maternally inherited symbionts (Sassera et al., 2008; Andreotti et al., 2011).

The abundant bacterial population in the tick ovaries and the negligible microbial community in the tick midgut indicate that the physiological role of the gut bacterial community, usually exercised in most metazoan, is accomplished in ticks by the obligate intracellular symbionts housed in the ovary. Indeed, it has been shown that genomes of both, *Midichloria mitochondrii* and *Coxiella* sp., encode genes that participate in the metabolic pathways for the biosynthesis of vitamins and cofactors suggested to be missed in the host blood (Sassera et al., 2011; Guizzo et al., 2017). *Coxiella* sp. in the ovary of *R. microplus* was demonstrated to play an important role in the tick development and reproductive fitness (Zhong et al., 2007; Guizzo et al., 2017; Zhang et al., 2017). Although the role of *M. mitochondrii* in biology of *I. ricinus* is likely similar to that of *Coxiella* sp. in *R. microplus*, further studies are needed to demonstrate its specific role.

In conclusion, using the quantification of the total 16S rDNA gene, we report here very low levels of the midgut microbiota in *I. ricinus* and *R. microplus*, without a core microbiome in *I. ricinus*. This corroborates the findings of a limited and unstable midgut microbiota in *Ixodes scapularis* (Ross et al., 2018). In contrast, several magnitudes higher levels of 16S rDNA were found in the ovaries of both tick species represented in *I. ricinus* by an endosymbiont *Midichloria* sp. The bacterial distribution in tick internal organs is an exception of the general biology of metazoans, characterized by a high ratio of a tick midgut/ovary microbiome.

## Data Availability Statement

Sequenced OTUs were deposited in the Genbank database under accession numbers MT255253- MT256050.

## Ethics Statement

This animal study was reviewed and approved by the Animal Protection Law of the Czech Republic No. 246/1992 Sb., ethics approval No. 25/2018. This study was approved by the Institute of Parasitology, Biology Centre CAS and Central Committee for Animal Welfare, Czech Republic (Protocol No. 1/2015). Ethics Committee on Animal Experimentation and were approved under the registry 14403/protocol 07.

## Author Contributions

MG conducted the experiments, analyzed the results, and wrote the manuscript. SN analyzed the results of 16S rDNA survey. MK and HF assisted with the experiments. JP, IS, PK, PO, and LZ conceived the experiments and wrote the manuscript. All authors reviewed the manuscript.

## Conflict of Interest

The authors declare that the research was conducted in the absence of any commercial or financial relationships that could be construed as a potential conflict of interest.

## Supplementary Material

Figure S1 Quantification of the 16S rDNA gene per the elongation *ef-1*α in the midgut **(A)** and ovary **(B)** of *Ixodes ricinus* and in the midgut **(C)** and ovary **(D)** of *Rhipicephalus microplus* during blood feeding on a vertebrate host. MG, midgut; OV, ovary; BF, blood fed; FF, fully fed; AD, after detachment; PEF, partially engorged female; FEF, fully engorged female. Each time-point represents the median of 7 biological replicates for *I. ricinus* and 6 for *R. microplus*. Error bars indicate standard deviation. Stars indicate statistically significant differences. ^*^*p* < 0.05; ^**^*p* < 0.01; and ^****^*p* < 0.0001.

Figure S2 Negative correlation between 16S rDNA gene in the midgut per the *ef-1*α and *Ixodes ricinus* weight during blood feeding on a vertebrate host. The results represent the median for 7 organs. Spearman's rank correlation coefficient = −0.88.

Table S1 Number of good quality 16S rDNA gene sequences obtained through bioinformatic analysis for the analyzed samples. MG, midgut; OV, ovary; BF, blood fed; FF, fully fed; AD, after detachment. Samples in yellow are above both sterility and reliability threshold. Samples in red were removed from the final analysis.

Table S2 Overview of the identified OTUs, and its classification into bacterial phylum and genera.
