# Supplementary figures and images for: Poor Unstable Midgut Microbiome of Hard Ticks Contrasts With Abundant and Stable Monospecific Microbiome in Ovaries
Source: Front Cell Infect Microbiol. 2020 May 8;10:211. doi: 10.3389/fcimb.2020.00211 (PMC7225584; doi:10.3389/fcimb.2020.00211)

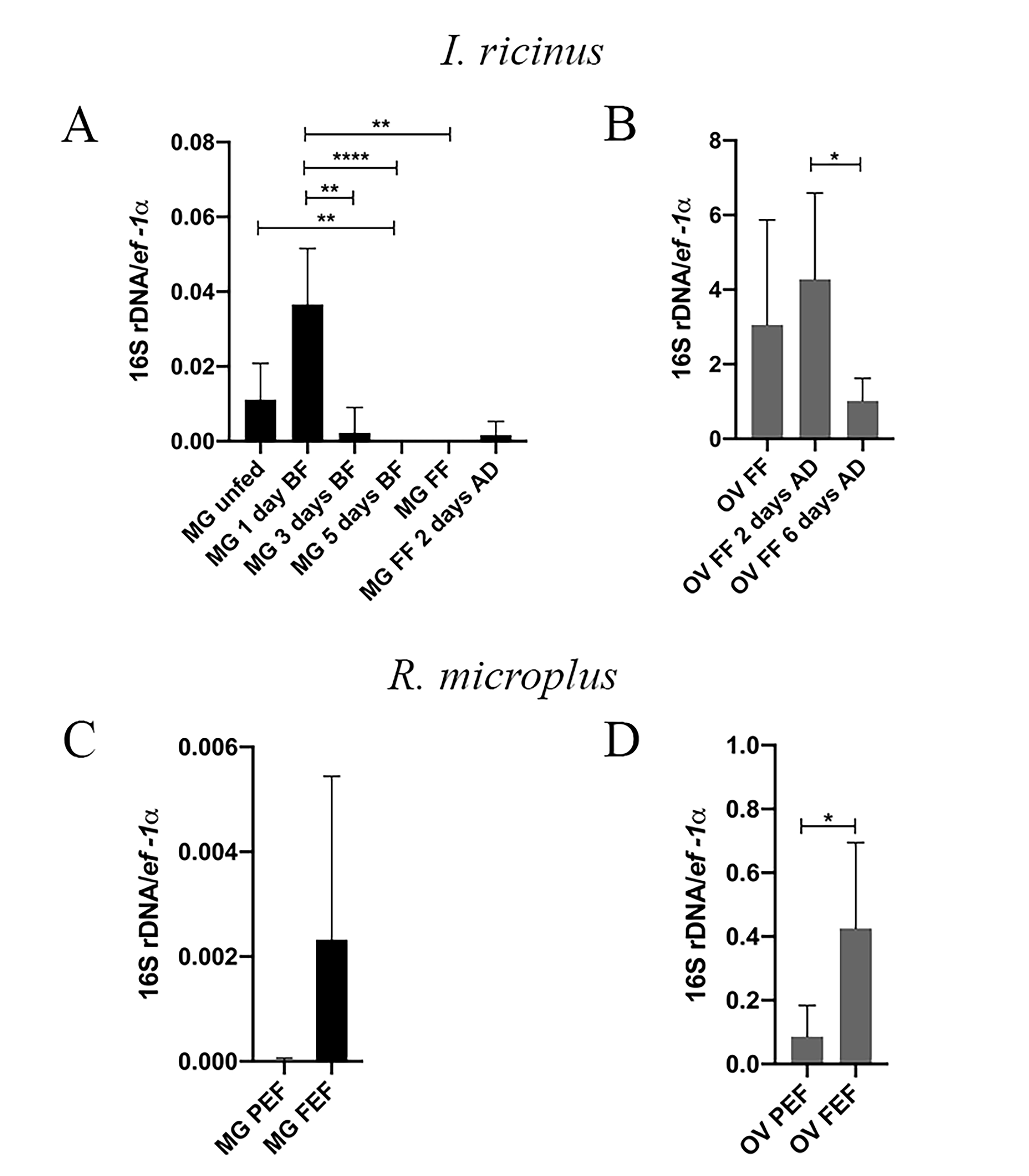

Supplement: Figure S1 — Quantification of the 16S rDNA gene per the elongation ef-1α in the midgut (A) and ovary (B) of Ixodes ricinus and in the midgut (C) and ovary (D) of Rhipicephalus microplus during blood feeding on a vertebrate host. MG, midgut; OV, ovary; BF, blood fed; FF, fully fed; AD, after detachment; PEF, partially engorged female; FEF, fully engorged female. Each time-point represents the median of 7 biological replicates for I. ricinus and 6 for R. microplus. Error bars indicate standard deviation. Stars indicate statistically significant differences. *p < 0.05; **p < 0.01; and ****p < 0.0001. [file Image_1.TIF]

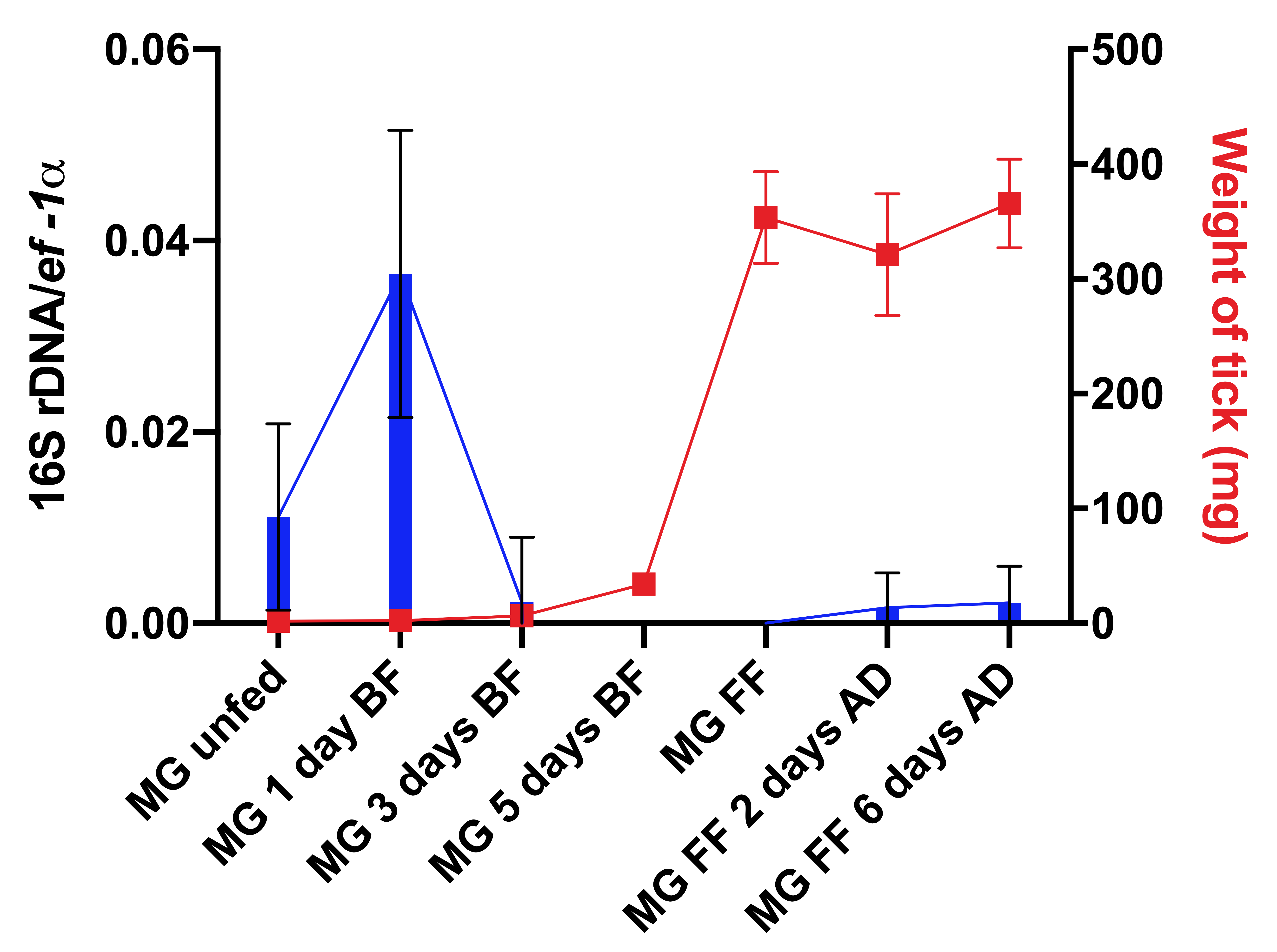

Supplement: Figure S2 — Negative correlation between 16S rDNA gene in the midgut per the ef-1α and Ixodes ricinus weight during blood feeding on a vertebrate host. The results represent the median for 7 organs. Spearman's rank correlation coefficient = −0.88. [file Image_2.TIF]
